# Wage-Earning Children

**Published:** 1920-04-17

**Authors:** 


### Wage-Earning Children.

The Committee on Wage-Earning Children are bringing pressure to bear on Labour members to amend their Bill providing for the forty-four hours week, especially where the employment of young persons and children is concerned. The Committee point out the need for special provisions to safeguard the young beyond those necessary for adults; for example, the Education Act will shortly compel young persons from fourteen to sixteen years to attend continuation classes for approximately eight hours per week. Without some special provision it would be possible for employers to compel young people to work the forty-four-hour week in addition to their attendance at class, thus giving them a fifty-two hours week, while adults had obtained forty-four. The Committee submit that a working week of thirty-six hours is sufficient for young persons, if in addition they are expected to attend continuation classes. It is also suggested that in the case of young people all overtime should be prohibited. They further propose that the Bill should contain a clause specially limiting the employment of all children under obligation to attend day school to fifteen hours in the week when the school is open on one or more days, and to twenty-five hours in the week when the school is not open.

